# Chemically Powered High-Aspect-Ratio Cilia with Tunable Biomimetic Motion

**DOI:** 10.1021/acsomega.6c05578

**Published:** 2026-10-17

**Authors:** Mohammad B. Uddin, Yaqeen Salatneh Ashqer, Suzanne Ahmed

**Affiliations:** Department of Nanoscience, Joint School of Nanoscience and Nanoengineering, 14616 University of North Carolina at Greensboro, 2907 E Gate City Blvd, Greensboro, North Carolina 27401, United States

## Abstract

Cilia are ubiquitous biological actuators that drive essential functions ranging from clearing contaminants from mammalian lungs to enabling locomotion and feeding in microorganisms. Their utility has inspired the development of artificial cilia. Yet while natural cilia operate through the conversion of chemical energy to mechanical motion, most artificial cilia made to date are externally powered using various fields. Most of these systems have also been of relatively low aspect ratios in comparison to their biological counterparts. In this work we report the synthesis and characterization of chemically powered, high-aspect-ratio artificial cilia with microscale diameters that exhibit individually autonomous, biomimetic oscillatory motion. Actuated using an oscillatory chemical reaction, the reaction catalyst is incorporated into the soft cilia hydrogel material where periodic oxidation and reduction of the catalyst induced periodic swelling and deswelling to generate motion. A customizable, 3D printed mold-based fabrication strategy is developed. The catalyst distribution, concentration, and cilium cross-sectional geometry are systematically varied across representative discrete levels to assess their influence on motion. The results show that motion is strongly governed by the interplay between catalyst loading and geometric asymmetry. Increasing the catalyst-containing stalk fraction and catalyst concentration enhances oscillation amplitude and motion regularity, whereas lower catalyst concentrations favor faster oscillatory dynamics. Geometric asymmetry enhances the speed, amplitude and periodic regularity of motion of these low Reynolds number structures. These findings demonstrate tunable motion in chemically driven artificial cilia and establish a framework for the design of biomimetic, chemically powered microscale actuators with potential applications in soft robotics.

## Graphical abstract

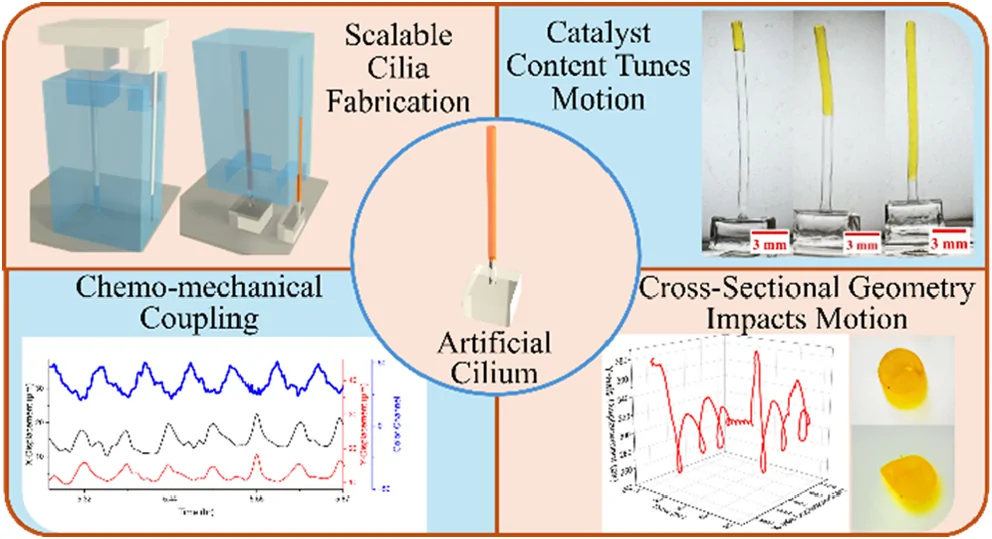

## 1 Introduction

Cilia are biological, hair-like structures that carry out critical functions in organisms as varied as single cellular prokaryotes and multicellular mammals.^1–8^ These slender, high-aspect-ratio appendages that produce fluid flow and locomotion, serve as a powerful source of inspiration for the design of synthetic active materials and actuators. Replicating their capabilities in artificial systems is an active area of research with applications in fluid pumping, particle transport and soft robotics.^9–36^ Another significant motivation for research into the synthesis and actuation of synthetic biomimetic systems is the shedding of light on the behavior of their biological counterparts to gain insight into their fundamental modes and mechanism of operation.^37–40^ Yet while natural cilia operate through biochemical processes that convert chemical energy into mechanical deflection in low Reynolds number environments, the majority of artificial cilia made to date have operated via external actuation powered using magnetic fields, electric fields, acoustic fields, pneumatics, or sweeping environmental changes such as changes in pH.^41–65^ Most recently the powering of macroscopic, individually autonomous artificial cilia by converting chemical energy to mechanical motion has been demonstrated by our group.^37^ In this system, a chemical reaction is used in conjunction with a soft gel that incorporates the reaction catalyst exclusively in the stalk of cilium and not the base, this distinct distribution within the 5:1 aspect ratio structure ensures the individual autonomy of the cilium motion with no basal contributions.^37^ In the trajectory toward attaining more biomimetic systems, in this work we report the first synthesis and motion characterization of chemically actuated, individually autonomous, high-aspect-ratio (20:1) artificial cilia with diameters on the microscale. Due to the difficulty in synthesis, handling and release of microscale, high-aspect-ratio cilia, the majority of reports of artificial cilia have been regarding those with aspect ratios below 10:1 with only a few of reports on cilia with microscale dimension with aspect ratios approaching approximately 20:1,^66–68^ with all being externally powered using magnetic or electromagnetic fields. In this work, we develop a synthetic approach that can reliably and reproducibility yield cilia with microscale diameters with aspect ratios that are 20:1 and potentially higher. Significantly these cilia are chemically powered akin to biological cilia.

These artificial cilia are autonomously actuated by the prototype oscillatory Belousov–Zhabotinsky (BZ) chemical reaction. This reaction is a nonlinear, oscillatory reaction that utilizes an organic chemical fuel.^69,70^ It was initially developed to mimic the biological Krebs cycle. A BZ responsive gel is utilized for the synthesis of the chemically powered artificial cilia. This material incorporates the organometallic BZ catalyst Ru­(bpy)_3_ ruthenium tris­(2,2′-bipyridine) covalently within the backbone of the stimuli-responsive polymer poly­(*N*-isopropylacrylamide) (PNIPAm). PNIPAm undergoes a conformational change in response to changes in temperature, swelling with water uptake and deswelling with water ejection at a critical transition temperature. At a constant temperature and with the BZ catalyst affixed to its backbone, the polymer undergoes the same conformational changes in response to changes in the oxidation state of the catalyst during the progress of the BZ reaction swelling in the more highly charged oxidized Ru^3+^ state and deswelling in the Ru ^2+^ state.^71,72^ As such this BZ-responsive gel can be used to mediate the transduction of chemical energy to mechanical motion. Indeed, this material, in conjunction with the BZ reaction, has a rich literature demonstrating the ability to actuate the mechanical swelling and deswelling of both tethered and untethered structures including those on the molecular scale to the macroscopic scale for BZ gels of varying structures including tubular, porous and corrugated materials.^73–92^

In this work we take a key step toward achieving more biomimetic artificial cilia by making high-aspect-ratio microscale cilia that are chemically powered to undergo sustained oscillatory motion. In utilizing the BZ reaction and the associated BZ material, high-aspect-ratio microscale cilia undergo the biomimetic oscillatory motion when submerged in a solution containing the BZ reactants. We systematically investigate the influence of catalyst content, including the catalyst-containing stalk fraction and the catalyst concentration, as well as the influence of the cross-sectional geometry of the cilia on the resulting dynamics. Our results reveal that both material composition and geometric design critically dictate the emergent motion of chemically powered cilia influencing oscillatory deflection amplitude, oscillatory regularity and cilium speed. These findings establish a framework for rationally designing chemically driven artificial cilia actuators with tunable performance with potential applications in autonomous soft robotics and fluid flow control.

## 2 Experimental Methods

Soft cilia were fabricated using an inverse molding approach based on 3D-printed master patterns (Figure 1). Customizable designs with defined dimensions were generated in AutoCAD and printed using an Elegoo Mars stereolithographic 3D printer. Following printing, the patterns were rinsed sequentially with water and isopropyl alcohol to remove excess resin, UV-cured for 60 min, and subsequently postcured in a convection oven overnight at 65 °C. This treatment yielded rigid, nonsticky resin-based cilia patterns suitable for mold fabrication. An Ecoflex silicone formulation was poured over the inverse mold and cured at room temperature overnight. After curing, the inverse mold was removed, resulting in a flexible, nonstick Ecoflex direct cilia mold. The cilia were designed to have a radius of 400 μm and a height of 16 mm, yielding an aspect ratio of 20:1.

**Figure 1:**
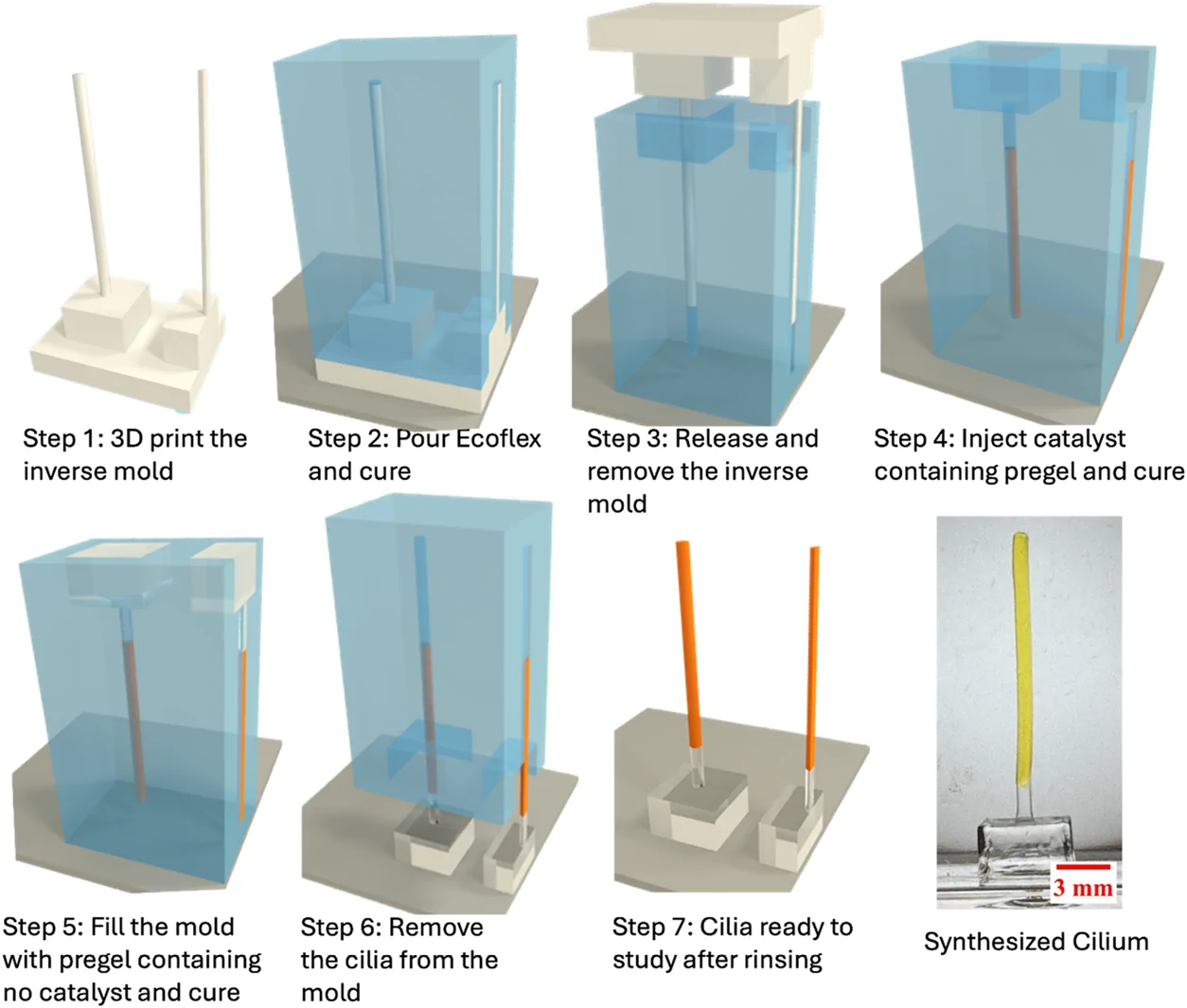
Schematic illustration of the synthesis process of artificial cilia. (1) Fabrication of a 3D-printed inverse mold pattern. A full cilium inverse mold is shown and an inverse mold cut down the cilium center is shown for illustrative purposes. (2) Preparation of a flexible mold by pouring an Ecoflex formulation over the pattern. (3) Release of the mold after Ecoflex is cured. (4) Injection of catalyst-containing pregel (orange) into the mold followed by UV curing. (5) Injection of catalyst-free pregel and subsequent UV curing. (6) Demolding of the fabricated cilium. (7) Schematic representation of the synthesized cilium. (8) Image of a synthesized cilium.

Two pregel formulations were prepared, one containing the catalyst and one catalyst-free. Both were composed of *N*-isopropylacrylamide (NIPAm) as the primary monomer, methylene bis­(acrylamide) as the cross-linker, and 2,2-dimethoxy-2-phenylacetophenone (DMPA) as the photoinitiator. In the catalyst-containing formulation, the organometallic catalyst used is Ruthenium tris­(2,2-bipyridine) [Ru­(bpy)_3_]. To covalently incorporate this catalyst into the polymer backbone of the gel, it is modified with a vinyl group while maintaining its catalytic properties, and hence the catalytic monomer used for the synthesis is (Ruthenium­(II)­(4-vinyl-4′-methyl-2,2′-bipyridine)­bis­(2,2′-bipyridine)­bis­(hexafluorophosphate), for conciseness the catalyst will be referred to as Ru­(bpy)_3_ in the remainder of the text. In a solvent mixture of 6.75 mL ethanol and 0.75 mL dimethyl sulfoxide (DMSO), the following reagents were dissolved: NIPAm (2.037 g), methylene bis­(acrylamide) (0.145 g), ruthenium monomer (0.013 g), and DMPA (0.24 g). The catalyst-free pregel employed the same formulation without the ruthenium compound.

Prior to polymerization, the pregel solutions were nitrogen-bubbled for at least 30 min to remove dissolved oxygen. The BZ-responsive, low viscosity, pregel solution containing the catalyst was slowly injected into the pores of the Ecoflex cilia mold using a thin needle (31 gauge) ensuring no bubbles build up or are trapped. The pregel is then UV-cured for 4 min within the transparent mold, thereby covalently incorporating the organometallic BZ catalyst into the PNIPAm polymer backbone. Subsequently, the noncatalyst pregel was injected into the same mold to form the remaining cilia structure and base, followed by another 4 min UV curing step. As such the catalyst containing portion of the cilium was exclusively in the stalk to ensure that ciliary motion was not due to basal deformation, but due to the independent autonomous fuel conversion in an individual cilium. The resulting hydrogel cilia were demolded without structural damage and subjected to graded ethanol/distilled water rinses over several days to remove unreacted monomers.

The dynamic response of the cilia was evaluated in a catalyst-free BZ solution composed of methyl malonic acid (0.0625 M), sodium bromate (0.084 M), and nitric acid (0.894 M). All experiments were performed at 24 ± 1 °C with 50 mL of BZ solution, ensuring complete submersion of the cilia. For each cilium type, four to six samples were evaluated. Ciliary motion was recorded simultaneously from top and dual side views oriented 90° apart. Videos were accelerated by a factor of 250× for visualization of motion. Motion tracking was performed using a custom MATLAB script, which extracted X and Y coordinates of cilia tips for each frame in both top and side views; and the properties of motion, such as speed, were determined after equilibration of the cilia within the BZ solution. The same code also quantified red, green, and blue (RGB) values of the sample, enabling determination of the catalyst redox state. To further illustrate wave propagation, RGB video files were decomposed into individual color channels. Unless otherwise noted, line/scatter traces of cilium motion (e.g., X-Y coordinates) show data from a single representative cilium per condition, while bar and summary plots report the mean ± SD across multiple cilia measured for that condition (*n* = 4).

## 3 Results

### 3.1 Cilium Motion

Upon immersion of the synthesized cilium in a solution of the Belousov–Zhabotinsky (BZ) reactants under acidic conditions, namely: methyl malonic acid reductant, sodium bromate oxidant, and nitric acid; periodic chemical waves propagate within the cilium. These redox-driven transitions are accompanied by the rhythmic beating of the cilium, resembling natural ciliary motion (Figure 2). Regular oscillations of the tip in both X and Y coordinates are correlated with the reversible redox cycling of the Ru­(bpy)_3_ catalyst as indicated by color, and the behavior is sustained for the full 48-h experimental time frame (Figure 2, Video S1).

**Figure 2:**
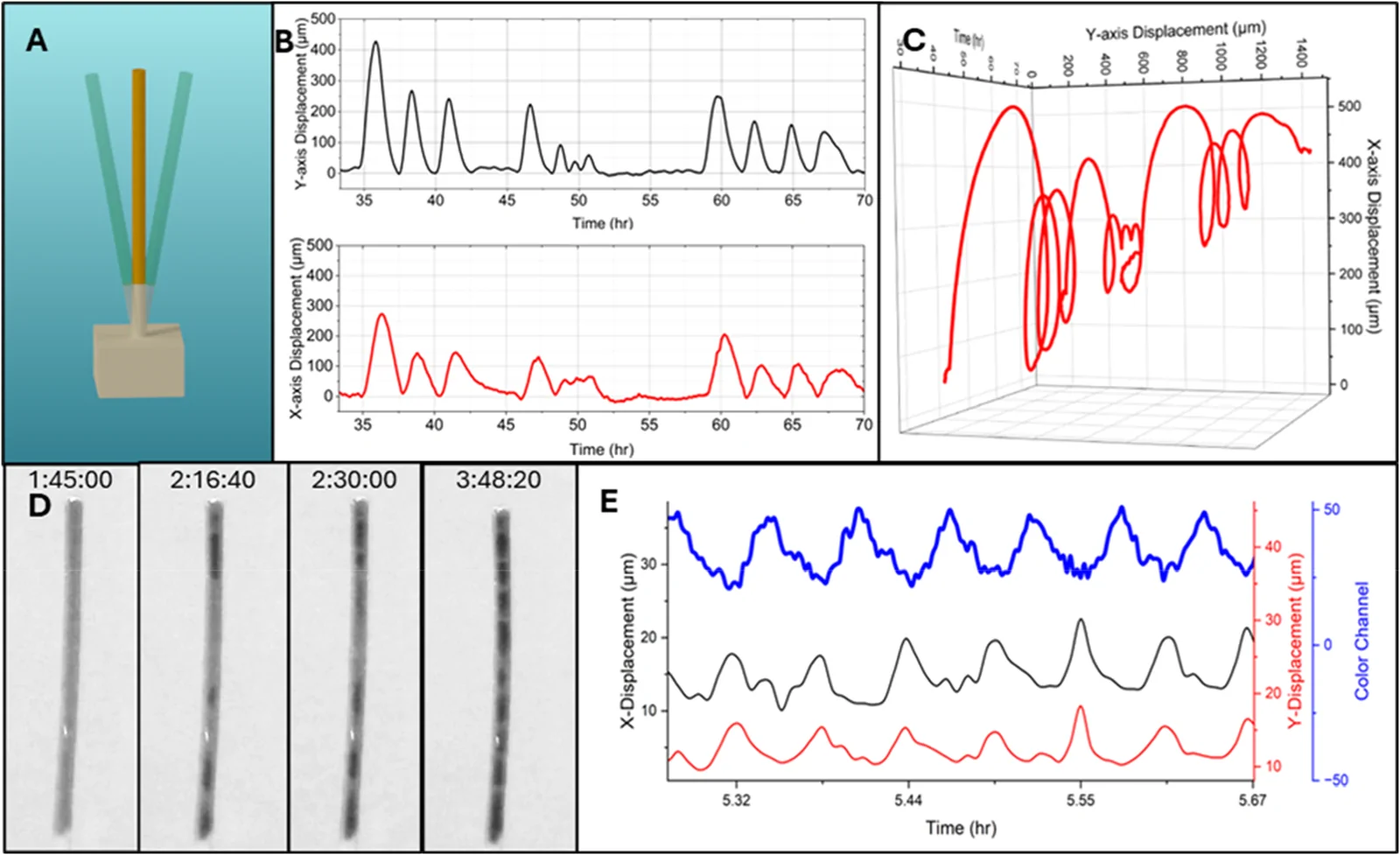
Motion and wave evolution of the cilium. (A) Schematic illustration of cilium motion while submerged in the BZ solution. (B) X and Y coordinates tracking data showing the oscillatory motion of the cilium tip. The large amplitude component of the motion is visualized. (C) Three-dimensional plot of the X–Y coordinates as a function of time, illustrating the tip trajectory. (D) Side views of the representative cilium showing the evolution of the chemical waves along the stalk.Time stamps are in hour: minutes: seconds format. The grayscale side view images are presented for improved perceptibility of the chemical waves (green is white, red is black). (E) Microscale oscillations synchronized with the chemical color change, blue color channel represented here.

The catalyst alternates between the reduced state (Ru^2+^) which has an orange color and the oxidized state (Ru^3+^) which has a green color, driving volume changes in the hydrogel network: the oxidized state enhances water uptake while the reduced state promotes release. Synchronization between tip displacement and fluctuations in color intensity (captured here in the blue channel) is observed, indicating a strong correlation between catalyst redox state and ciliary motion; this relationship confirms that the observed beating arises from chemo-mechanical cycling driven by redox-induced swelling and deswelling, consistent with the established mechanism of motion in previously reported macroscopic BZ gels.^37,71,72,86–88^

The chemically actuated artificial cilia studied operate in the low Reynolds number regime. Calculating the Reynolds number from eq ([Disp-formula eq1])

1 Re=lρvη

where *l* is the characteristic length, taken to be the diameter of the equivalent volume sphere of the 400 μm radius and 16 mm tall cylindrical cilium, *v* is the average speed determined to be 11 μm/min, ρ is the density of the solution and η dynamic viscosity, both estimated to be that of water for the aqueous solution, the Reynolds number is 4 × 10^–4^ which is orders of magnitude less than 1.

The cilium tip follows an elliptic trajectory where the forward and return strokes of each cycle do not perfectly overlap; instead, the tip traces slightly displaced paths that together outlined a closed ellipse (Figure 2, Video S1). This asymmetry is most likely attributable to local chemical gradients generated by the depletion of reactants and accumulation of products along the forward trajectory, which cause the backward motion to follow a subtly shifted path due to a chemotactic effect as previously experimentally and computationally identified for tethered and untethered BZ systems.^37,86,93^ The overall motion consists of two superimposed oscillatory modes: a large-amplitude component reaching several millimeters (Figure 2(B)) and a fast, small-amplitude component on the order of tens of microns (Figure 2(E)). This latter pattern is strongly correlated with the propagation of waves along the cilium stalk (Figure 2 D, E). These two modes can be understood in terms of the way waves propagate through BZ gels of different sizes,^94^ for millimeter scale BZ gels, such as the length of the cilium stalk, distinct wave patterns appear and their propagation and evolution are visible; in the case of dimensions on the microscale; such as that of the diameter, a global pulsing mode occurs, this would encompass the full cilium due to the microscale diameter along its length, and hence is the most likely contributor to the larger amplitude oscillations observed. Waves along the cilium stalk evolve with time as is common for BZ waves within gels, these waves can initiate from one end of the cilium, such as the base, from the two ends and meet in the center or meet off center (Figure 2D, Video S2).

### 3.2 Influence of the Percentage of the Stalk Containing Catalyst on Ciliary Motion

To investigate the influence of catalyst-containing stalk fraction on motion, cilia were fabricated with 20%, 50%, and 80% of their stalk length containing the Ru­(bpy)_3_ catalyst (Figure 3A). The remaining portion of each structure was composed of catalyst-free hydrogel, forming the flexible base.

**Figure 3:**
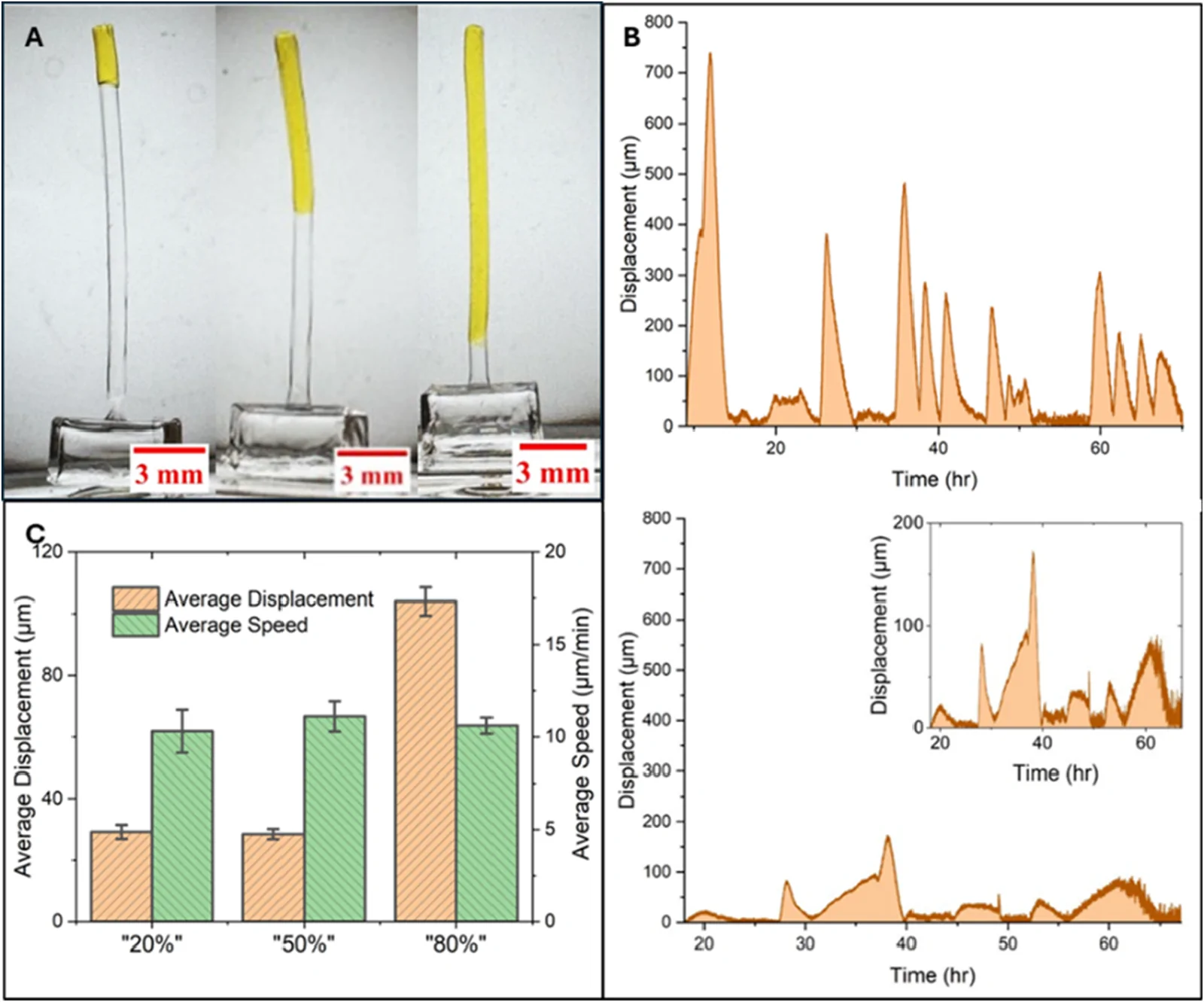
Effect of Catalyst-Containing Stalk Fraction on Ciliary Motion. (A) Static, prereaction images of artificial cilia with the catalyst-containing region occupying 20%, 50%, and 80% of the stalk length. (B) Comparison of oscillation displacement amplitude and regularity between representative cilia with 80% catalyst-containing region (top) and 20% catalyst-containing region (bottom) on the same scale, with the inset showing a magnified view of the oscillation profile. (C) Plot of the average tip speed and the average tip displacement of cilia with increasing catalyst-containing fraction.

Analysis of tip displacement reveals a strong dependence on catalyst-containing stalk fraction. Cilia with larger catalyst fractions (80%) display highly regular oscillations, with displacement trajectories repeating consistently over time (Figure 3B). By contrast, cilia with lower catalyst containing volume (20%) exhibit more irregular displacement profiles, with noticeable variations in amplitude and frequency across successive cycles. These observations suggest that a greater proportion of active catalyst enhances the stability and strength of chemo-mechanical coupling producing more reproducible beating patterns. This increased stability likely arises from the larger percentage of active, motion-generating fraction of the gel relative to the passive portion of the stalk, resulting in more effective self-reinforcement of the chemo-mechanical response during each oscillation cycle.

Interestingly, the average tip speed remains relatively constant across all three groups, this can be explained by the fact that speed is correlated with oscillation period which is constant for a fixed concentration of catalyst regardless of catalyst volume (Figure 3C). However, the average displacement amplitude increases with greater catalyst-containing stalk fraction. In particular, the 80% samples achieved the largest deflections, while the 20% samples were limited to smaller displacements. This trend demonstrates that while the oscillation rate is preserved, the range and reliability of motion are governed by the volume fraction of catalyst incorporated into the cilium.

### 3.3 Influence of Catalyst Concentration on Motion

To examine the influence of catalyst loading on the chemo-mechanical response of BZ-driven artificial cilia, two sets of samples were fabricated with different Ru­(bpy)_3_ catalyst contents: 8 × 10^–2^ mol % catalyst, that will be referred to as (Ru100), and one containing 75% of that amount at 6 × 10^–2^ mol % catalyst, that will be referred to as (Ru75), while maintaining identical catalyst-containing stalk fractions and dimensions. Calculations in supporting information. The comparison of these systems reveals distinct differences in their oscillatory behavior, period, and mechanical performance.

In evaluating the oscillation period over time, it was found that Ru75 cilia exhibit a shorter oscillation period compared to Ru100 cilia, demonstrating a faster redox cycling rate. On average, a 15% reduction in the oscillation period is observed for samples with a lower mol % of catalyst. Representative cilium samples are shown in Figure 4. Increases in oscillatory period with the increase in catalyst concentration has been reported for both BZ gels as well as BZ solutions and can be attributed in large part to catalyst turnover with larger concentrations taking longer durations.^95–97^

**Figure 4:**
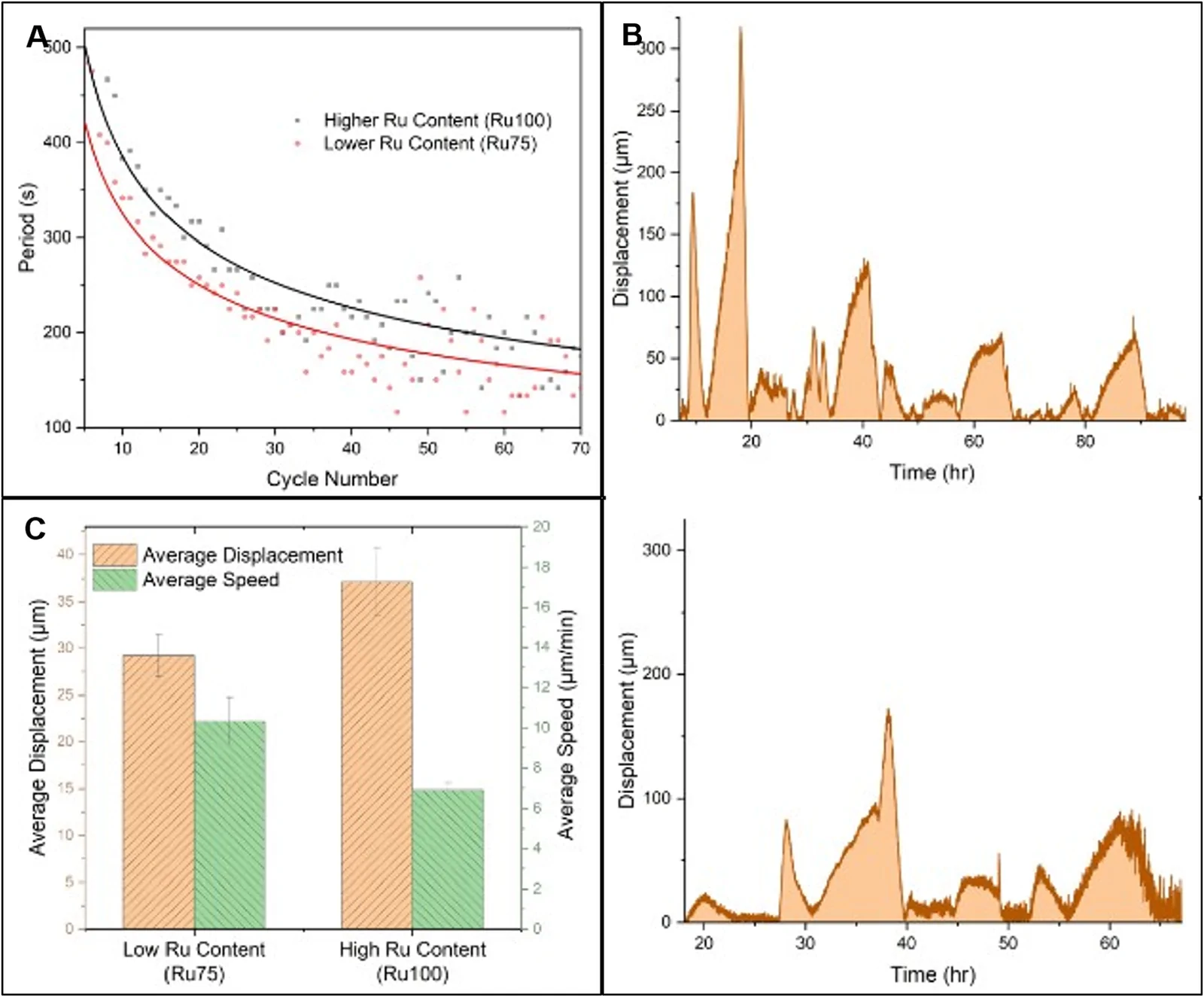
Influence of catalyst concentration on ciliary motion. (A) Oscillation period for representative cilia over time where each point represents the period for consecutive cycles. The fitted curves indicate that the Ru100 sample exhibits a higher average period compared to the Ru75 sample. (B) Comparison of oscillation regularity between cilia with higher Ru content (Ru100, top) and lower Ru content (Ru75, bottom) at a fixed 20% catalyst-containing stalk fraction. (C) The Ru100 sample shows a lower average tip speed but a higher average oscillation amplitude compared to Ru75.

Quantitative analysis of cilium motion shows that Ru100 samples have a lower average speed, approximately 33% less than Ru75, which can be attributed to their longer oscillation period and slower chemo-mechanical actuation. However, the amplitude of motion for Ru100 is approximately 25% higher, suggesting that a higher catalyst density enhances the magnitude of swelling and contraction cycles despite the reduced frequency. This implies a trade-off between actuation rate and displacement magnitude governed by the extent of catalyst incorporation within the polymer matrix.

The regularity of motion also varies substantially between the two concentrations for a fixed 20% catalyst-containing stalk fraction (Figure 4B). The Ru100 cilia exhibit more stable and periodic oscillations over time, with little fluctuation in displacement amplitude or phase, indicating well-synchronized redox-mechanical coupling. In contrast, Ru75 cilia show greater variability and less consistent motion, possibly due to weaker chemical and mechanical coupling at lower catalyst loading.

Collectively, these results highlight that catalyst concentration serves as a critical tuning parameter for controlling the oscillation kinetics and mechanical output of BZ-responsive cilia. While a reduced catalyst concentration enhances oscillation frequency and speed, higher Ru­(bpy)_3_ catalyst content enhances the amplitude and regularity, offering a design trade-off between responsiveness and stability in chemically actuated soft systems.

### 3.4 Influence of Cilium Cross Sectional Shape on Motion

To elucidate the influence of geometry on the chemo-mechanical performance of BZ-responsive cilia, two distinct designs with circular and drop-shaped cross sections were fabricated. The cross-sectional area was kept constant for both geometries to ensure that differences in motion originated purely from shape effects rather than volume. Cilia were fabricated to have an identical 6 × 10^–2^ mol % catalyst concentration at a fixed 20% catalyst-containing stalk fraction. Despite this controlled design, the two geometries exhibited markedly different oscillatory characteristics when immersed in the BZ solution.

The drop-shaped cilia consistently demonstrated superior dynamic behavior compared to their circular counterparts. Quantitative analysis of tip displacement reveal that the drop-shaped configuration achieves both higher average speed and greater oscillation amplitude throughout the 48 h observation period, with the average speed increasing by approximately 90% and the average amplitude increasing by about 70% relative to the circular cilia (Figure 5, Video S3). The enhanced performance is attributed to the asymmetric geometry of the drop-shaped design, which may influence the distribution and propagation of redox-induced swelling along the cilium length, potentially contributing to the stronger and faster bending responses observed. Additionally, for a low Reynolds number system asymmetry enhances displacement as per the scallop theorem,^98^ which states that for a net displacement to occur symmetry must be broken. While symmetry is broken for the circular cilia in the propagation of waves within the structure, as well as in the intrinsic asymmetry of the cilium due to it being tethered at one end, and since a portion of the stalk and base contain no catalyst inserting compositional asymmetry, the addition of a cross sectional shape asymmetry in the design of the cilium, as with the drop-shaped cilia, increases the level of asymmetry resulting in a greater displacement.

**Figure 5:**
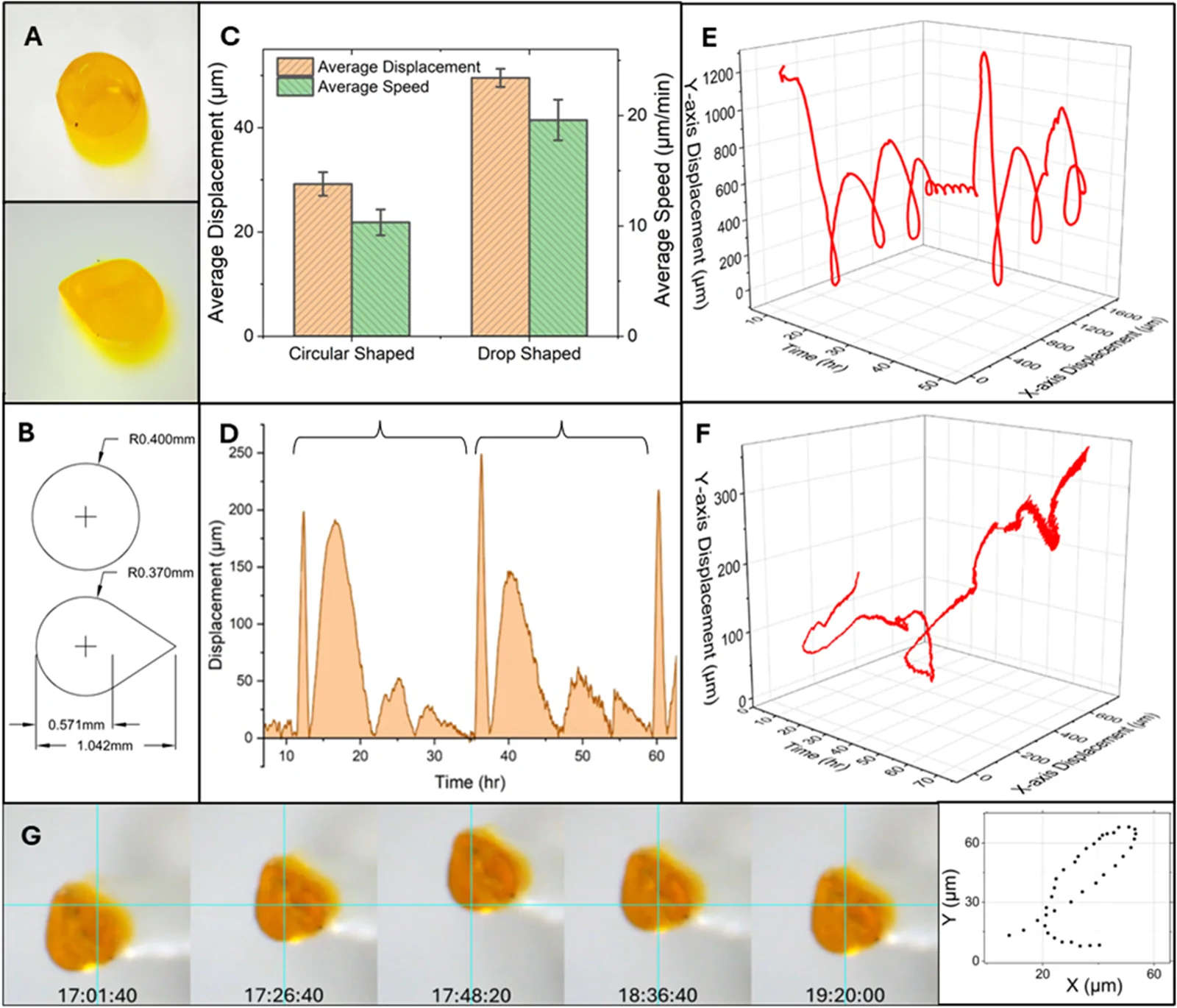
Influence of cilia shape on ciliary motion. (A) Top view of circular-shaped (top) and drop-shaped (bottom) representative cilia. Samples with a 6 × 10^–2^ mol % catalyst at 20% catalyst-containing stalk fraction of each cross section were made. (B) Dimensions of the different shapes, each with a constant surface area of 0.503 mm^2^. (C) Both the average tip displacement and average speed increase for drop-shaped cilia compared to circular-shaped cilia. (D) Displacement data for the drop-shaped sample shows greater oscillation regularity. (E) Three-dimensional plot of the tip motion of drop-shaped cilia. (F) Three-dimensional plot of the tip motion of circular-shaped cilia. (G) Time-lapse montage of motion of a drop-shaped artificial cilium and the corresponding tracked tip trajectory, showing a directional displacement pattern.

In addition to amplitude and speed, the motion of drop-shaped cilia exhibit greater regularity and periodicity. The displacement–time profiles show highly repetitive cycles with minimal variation between successive oscillations, by contrast, the circular cilia displayed less regular motion, with fluctuations in both amplitude and directionality. The drop-shaped cilia also exhibit a more directional, linearly oriented beating pattern, resembling a more efficient effective recovery stroke similar to biological cilia. This anisotropic motion is likely a direct consequence of the drop-shaped cross-section, which induces uneven stress distribution during the periodic redox-driven expansion and contraction of the gel (Figure 5).

Collectively, these results demonstrate that the drop-shaped geometry enhances both the speed, displacement amplitude and regularity of chemo-mechanical actuation in BZ-driven soft cilia. This suggests that geometric asymmetry can serve as a key design parameter for optimizing propulsion and fluid manipulation in chemically powered microactuators.

### 3.5 Cilium Propulsive Force

The drag force on a system in the low Reynolds number regime (Re < 1) corresponds to its propulsive force. An order of magnitude estimate of the drag force on the cilium can be obtained by approximating it as a slender cylinder and applying eq ([Disp-formula eq2]) below.^9^

2 $$F_{drag}=\frac{4\pi \mu \mathrm{lv}}{\ln\left(\frac{l}{r}\right)}$$

where *l* and *r* are the length and radius of the cilium, μ is the fluid dynamic viscosity, and *v* is the speed of the cilium. The drag force acting on the cilium is calculated to be 9 × 10^–12^ N (9 pN) when moving at a speed of 11 μm/min. This drag force is approximately 2 orders of magnitude smaller than the drag experienced by macroscale cilia with smaller aspect ratio,^37^ as would be expected for the more slender profile. For these high-aspect-ratio circular cilia (80% stalk fraction), the maximum deflection is approximately one body length (defined here as the cilium diameter of 800 μm). By comparison, reported maximum deflections for comparable BZ-driven hydrogel actuator systems are typically in the range of 0.15–0.3 body diameters.^37,86^ While externally actuated forced systems, especially those that are magnetically actuated, can achieve larger deflections at high frequencies they require external apparatus for operation which can reduce their potential utility. Another distinguishing feature of these biomimetic systems is the use of a stimuli-responsive material for their synthesis. BZ-responsive hydrogels are responsive to light, temperature and the chemical composition of the actuating solution.^81^ These qualities open the door for the control of their motion using different stimuli in future studies. Additionally, the biomimetic nature of chemically actuated autonomous systems may allow their use as model platforms for the study of the operation of their biological counterparts.^37^

## 4 Conclusion and Discussion

This study reports the synthesis and motion characterization of high-aspect-ratio microscale cilia that are chemically powered autonomous entities. This study demonstrates that the dynamic behavior of the Belousov–Zhabotinsky (BZ) reaction driven artificial cilia is strongly influenced by the spatial distribution and the concentration of the Ru­(bpy)_3_ catalyst, as well as the cilium’s geometric shape. Through a systematic variation of cilium parameters and investigation of motion characteristics, clear trends emerged linking catalyst loading and geometry to oscillation frequency, amplitude and motion regularity.

Cilia with larger catalyst-containing stalk fractions exhibit greater oscillation amplitudes and improved periodic regularity in beating cycles. The enhanced regularity in the 80% catalyst- containing stalk fraction samples in comparison to 20% ones, highlights the role of increased active material in maintaining a robust chemo-mechanical actuation coupling. However, the oscillation frequency remains largely unaffected by catalyst volume, suggesting that the volume fraction predominantly governs the mechanical output rather than the oscillation kinetics.

The catalyst concentration-dependent experiments reveal a complementary trade-off between catalyst loading and actuation behavior. Samples with lower catalyst concentrations display shorter oscillation periods and correspondingly faster tip motion, whereas the samples with higher catalyst concentrations, despite slower oscillations, produced higher displacement amplitudes and more regular motion. These findings indicate that the rate of redox cycling, and therefore the chemo-mechanical frequency can be fine-tuned through dilution of the catalyst, while the overall stability and regularity of oscillations benefit from higher catalyst concentrations. Such control over oscillation kinetics and mechanical amplitude provides a valuable design handle for tailoring ciliary performance for specific applications, where either speed or amplitude may be prioritized.

Geometric effects further amplified these trends. The comparison between circular and drop-shaped cilia revealed that shape asymmetry strongly enhances the speed, regularity and displacement amplitude of motion. The drop-shaped cilia not only achieved higher average speeds (∼90% increase) and larger amplitudes (∼70% increase) relative to circular cilia but also exhibited more regular beat patterns. The anisotropic geometry likely induces nonuniform stress propagation within the gel, leading to more effective translation of local chemical oscillations into directional motion. This resemblance to the effective–recovery stroke of natural cilia underscores the importance of geometric asymmetry in achieving biomimetic actuation.

Taken together, the results demonstrate that the interplay between catalyst concentration, distribution, and geometry governs the chemo-mechanical coupling efficiency in BZ-driven soft actuators. High catalyst content enhances amplitude and regularity, while moderate concentrations favor faster oscillations. Geometric asymmetry, meanwhile, enhances displacement, speed and regularity of motion, improving actuation performance. These findings establish a framework for designing next-generation autonomous soft actuators, where precise control over geometry and catalytic composition enables tunable rhythmic motion for potential applications in microfluidic transport, self-propelled microrobots, and bioinspired pumping systems. Future work incorporating external stimuli could enable on-demand control of wave propagation and hence motion, moving this platform toward fully programmable ciliary actuation.

## Supplementary Material









## Notes

Circular Cilia Motion (MP4)
Wave Pattern Evolution (MP4)
Drop Shaped Cilia Motion (MP4)
Descriptions of Supporting Information Videos and calculations of catalyst concentrations (PDF)
