# Supplementary material for: Chemically Powered High-Aspect-Ratio Cilia with Tunable Biomimetic Motion
Source: ACS Omega. 2026 Oct 17;11(43):66438–48. doi: 10.1021/acsomega.6c05578 (PMC13736972; doi:10.1021/acsomega.6c05578)
Supplement: Supplementary file 1 [file ao6c05578_si_004.pdf]

Supporting Information for

Chemically Powered High-Aspect-Ratio Cilia with Tunable Biomimetic Motion

Mohammad B. Uddin, Yaqeen Salatneh Ashqer, Suzanne Ahmed*

[1]Department of Nanoscience, Joint School of Nanoscience and Nanoengineering, University of North Carolina at Greensboro, 2907 E Gate City Blvd, Greensboro, NC 27401
Corresponding Author: * saahmed2@uncg.edu

Video S1. Circular Cilia Motion

Oscillatory motion of circular-shaped artificial cilia in Belousov–Zhabotinsky (BZ) solution. The cilium is positioned at the center of a transparent acrylic chamber (41mm×41mm×41mm). The first segment shows the top view of the cilium, followed by a side view illustrating the reciprocating motion. A magnified view of the cilium tip is also provided to clearly visualize the oscillatory movement (Side View).

Video S2. Wave Pattern Evolution

Oxidation wave evolution in two cilia with the catalyst-containing region occupying 80% of the stalk length. The right panel shows the original recording, while the left panel displays the blue-channel-only view, where the propagation of chemical waves is more clearly visible.

Video S3. Drop Shaped Cilia Motion

Oscillatory motion of drop-shaped artificial cilia in BZ solution. The first and second segments show the top and side views of a drop-shaped cilium, respectively. The third segment presents the top view of another drop-shaped cilium undergoing oscillatory motion in BZ solution.

$Ru(bpy)_3$ monomer mol % calculation:

The catalyst concentration is reported as mol % of $Ru(bpy)_3$ monomer relative to the total moles of all monomeric/crosslinking species used in the pre-gel formulation (i.e., NIPAAm + BIS + Ru monomer combined), calculated as:

$$\text{mol \% Ru} = \frac{\text{mol Ru monomer}}{(\text{mol NIPAAm} + \text{mol BIS} + \text{mol Ru monomer})} \times 100$$

For the Ru100 formulation:

Component	Mass (g)	MW(g/mol)	Moles
NIPAAm	2.037	113.16	0.01801
BIS	0.145	154.17	0.00094
Ru monomer	0.013	834.78	0.0000156

Total moles = 0.01897 mol

Mol % Ru = (0.0000156/ 0.01897) × 100 **≈ 8×10^{-2} mol %**

For the Ru75 formulation:

Component	Mass (g)	MW(g/mol)	Moles
NIPAAm	2.037	113.16	0.01801
BIS	0.145	154.17	0.00094
Ru monomer	0.0098	834.78	0.0000117

Total moles = 0.01896 mol

Mol % Ru = (0.0000117/ 0.01896) × 100 **≈ 6×10^{-2} mol %**
